# Comparison of Transforaminal Magnesium Sulfate with Steroid Injection in the Management of ‎Radicular Back Pain: A Randomized Double-Blinded Clinical Trial Study

**DOI:** 10.5812/aapm-148739

**Published:** 2024-09-08

**Authors:** Farnad Imani, Kambiz Sadegi, Poupak Rahimzadeh, Mania Kaveh, Mahnaz Narimani, Seyed-Hossein Khademi

**Affiliations:** 1 Pain Research Center, Department of Anesthesiology and Pain Medicine, School of Medicine, Iran University of Medical Sciences, Tehran, Iran; 2 Department of Anesthesiology, Zabol University of Medical Sciences, Zabol, Iran; 3 Department of Gynecology and Obstetrics, Zabol University of Medical Sciences, Zabol, Iran; 4 Department of Anesthesiology, Tehran Medical Science, Islamic Azad University, Tehran, Iran; 5 Department of Anesthesiology, School of Medicine, Mashhad University of Medical Sciences, Mashhad, Iran

**Keywords:** Radicular Back Pain, Epidural Injection, Transforaminal Space, Magnesium Sulfate, Steroid

## Abstract

**Background:**

This study compares the effects of transforaminal magnesium sulfate injection versus other methods for managing radicular back pain, highlighting its potential for improved pain relief and functional outcomes.

**Methods:**

This randomized, double-blind clinical trial involved 30 patients with radicular back pain who were randomly assigned to receive either transforaminal magnesium sulfate or triamcinolone injection. Primary outcomes were pain intensity and functional disability, assessed using the Visual Analogue Scale (VAS) and Oswestry Disability Index (ODI), respectively. These were evaluated at five time points: Before the injection, 2 weeks, 1 month, 3 months, and 6 months after the injection. Secondary outcomes included drug-related adverse events within the six-month follow-up period.

**Results:**

Baseline characteristics were not significantly different between the two study groups. Compared to pre-injection measures, post-injection pain intensity and functional disability were significantly reduced in both groups at all time points (P < 0.001). At all postoperative evaluations, pain intensity and functional disability were lower in the magnesium sulfate group compared to the steroid group (P < 0.001). No drug-related side effects were recorded in either group.

**Conclusions:**

For patients with radicular back pain, transforaminal magnesium sulfate injection appears to be an effective and safe alternative to transforaminal steroid injection.

## 1. Background

Radicular back pain, also known as lumbosacral radiculopathy, is among the most common types of back pain, with a prevalence of approximately 3 to 5% in the general population (1). The primary underlying cause of radicular back pain is nerve irritation, typically due to compressive forces at various points along the spinal column (2). Other potential causes of radiculopathy include disc bulging or herniation, spondylolisthesis, facet or ligamentous hypertrophy, and less commonly, infectious or neoplastic processes (3).

Current guidelines recommend conservative management as the initial treatment approach for radicular back pain. This management typically involves education, exercise, manual therapy, and non-steroidal anti-inflammatory drugs (NSAIDs) (4). If conservative measures are ineffective, pain injections are considered as a second-line treatment (4). These injections deliver medication directly into the epidural space via various techniques such as caudal, interlaminar, or transforaminal approaches (5), and have been shown to provide long-term symptom relief (6).

Epidural injections generally consist of a combination of anti-inflammatory medications and long-lasting anesthetic agents. Glucocorticoids, such as triamcinolone, are commonly used anti-inflammatory medications in these injections for treating radicular back pain (3). However, epidural steroid injections can be associated with various potential adverse effects, including facial and chest flushing, acute neurological symptoms, sleep disturbances, water retention, metabolic and endocrine changes, and, in rare cases, spinal cord infarction (2, 7, 8). Consequently, there is significant interest in identifying safe and effective alternatives for epidural injection in the treatment of radicular back pain.

Magnesium sulfate, an antagonist of the N-methyl-d-aspartate receptor, helps prevent central sensitization and attenuates preexisting pain hypersensitivity. It has been reported as a safe and effective treatment for neuropathic pain when administered orally or parenterally (9). Recently, magnesium sulfate has garnered attention for its potential role in managing radicular back pain, particularly when combined with a local anesthetic and steroid, leading to improved pain relief and quality of life (10).

Transforaminal steroid injection is one method for managing lumbar radicular pain. It has gained popularity due to its specific advantages, including increased specificity, lower injection volume, and direct targeting of the primary pathological site (11).

To the best of our knowledge, the effects of epidural magnesium sulfate injection as an alternative to epidural steroid injection on pain intensity and functional disability in patients with radicular back pain have not been previously investigated. 

## 2. Objectives

This study aims to compare the safety and effectiveness of epidural magnesium sulfate versus epidural steroid (triamcinolone) injection in treating radicular back pain.

## 3. Methods

### 3.1. Ethics

This study was approved by the review board of Iran University of Medical Sciences under code IR.IUMS.REC.1398.1335. The study protocol was registered with the Iranian Registry of Clinical Trials under code IRCT201312037984N12. Written informed consent was obtained from all patients prior to their participation in the study.

### 3.2. Study Design

In a randomized double-blind clinical trial conducted in the operating room and pain department of Hazrat Rasool Akram Hospital in Tehran, 30 patients with severe lumbar radicular pain were included. The inclusion criteria were: Age between 20 and 70 years, radicular pain lasting at least six weeks, evidence of nerve root involvement (such as intervertebral disc herniation or foraminal stenosis) on CT or MRI, Visual Analogue Scale (VAS) score greater than 4, American Society of Anesthesiologists (ASA) class I or II, and non-responsiveness to conservative (medical and physical) treatments. Exclusion criteria included neurological deficits, coagulation abnormalities, local or systemic infections, severe psychiatric disorders, history of spinal surgery, vertebral deformities such as scoliosis, pregnancy, drug abuse, cancer, allergy to study drugs, and immunosuppressive disorders such as HIV.

**Figure 1.**
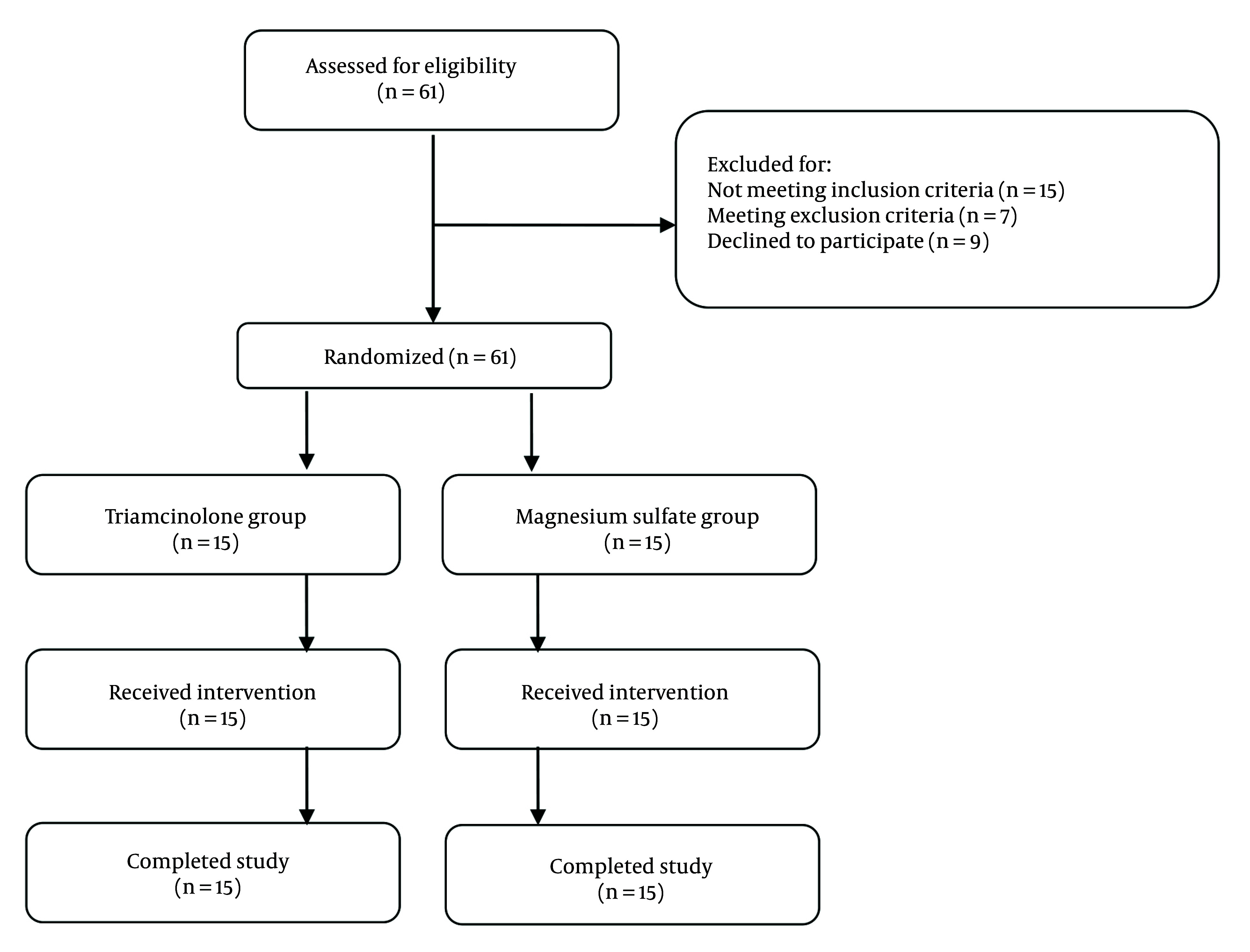
Flow diagram of the study

Eligible patients were randomly assigned to one of two study groups: Epidural steroid (triamcinolone) injection or epidural magnesium sulfate injection. The study flow diagram is shown in Figure 1. No changes were made to the study protocol after the commencement of the experiment (Figure 1). 

### 3.3. Intervention

The patient was positioned accurately, and a lumbar transforaminal block was performed under sterile conditions using local anesthesia with 2% Lidocaine. Under fluoroscopic guidance, a 16-gauge introducer needle was inserted beneath the intersection of the transverse process and the pedicle. After removing the stylet, a blunt radiofrequency (RF) needle, 10 cm in length with a 1 cm active tip, was inserted to the target site in the posterior cephalic foramen. The correct location of the needle was confirmed using fluoroscopy. 

Next, 1 cc of contrast agent (Visipaque 270) dissolved in non-ionized water was injected, and its distribution along the nerve path was observed. Subsequently, the injectable substance was administered: 20 mg of Triamcinolone (Triamcinolone acetonide, Exir, Iran) in 4 mL of 0.2% Ropivacaine (Ropivacaine, Molteni, Italy) for the Triamcinolone group, and 150 mg of Magnesium Sulfate (McGuff Pharmaceuticals) in 4 mL of 0.2% Ropivacaine for the Magnesium group. In cases of two-level involvement, the same dosage was administered at the additional level. Proper injection was confirmed with another fluoroscopic image. 

After needle removal, the surgical area was cleaned, and a sterile bandage was applied. The patient was then transferred to the recovery room and monitored for two hours. Once the patient’s condition was stable, they were discharged with oral pregabalin (Lyrica, Pfizer, Germany) 75 mg to be taken before bedtime. For patients with a VAS score greater than 3, acetaminophen 500 mg (Zagros Pharmed Pars Pharmaceuticals, Iran) was administered every six hours.

### 3.4. Outcome Measures

The primary outcomes included lumbar pain and function. Lumbar pain was assessed using the Visual Analog Scale (VAS), which measures the distance on a 10-cm line with two endpoints: No pain (VAS = 0) and worst pain (VAS = 10). Lumbar function was evaluated using the Oswestry Disability Index (ODI), with scores ranging from 0 to 100, where 0 indicates no disability and 100 indicates the worst disability. The Persian translation of the ODI, which has been validated and shown to be reliable in a previous study (12), was used. Visual Analog Scale and ODI scores were assessed at five time points: Before the intervention, two weeks after the intervention, and one, three, and six months post-intervention.

Secondary outcomes included post-intervention complications related to the injection substance, which were recorded throughout the study period.

### 3.5. Randomization and Blinding

Using the four-block method of randomization, patients were randomly divided into two equal groups of 15 subjects each. To ensure the study was double-blind, neither the patients nor the researchers were aware of the type of intervention being administered. Thirty random numbers, including 15 even and 15 odd numbers, were generated using Excel software and placed in sealed opaque envelopes. These envelopes were given to an independent assistant who was not directly involved in the study. When a patient was referred for the injection, the envelope was opened to reveal the number. Based on whether the number was odd or even, the patient received either a Triamcinolone or Magnesium Sulfate injection. Neither the patients nor the researchers knew which type of intervention the participants were receiving.

### 3.6. Sample Size

In the study by Awad et al., the mean VAS score of patients one week after the intervention was 19.8 ± 6 in the steroid group and 10 ± 4.2 in the steroid combined with magnesium group (10). Based on these data, a power of 95%, a type I error rate of 0.05, and an effect size of 1.89, it was determined that 10 patients in each study group was sufficient to conduct this comparative study of two independent groups.

The sample size was calculated using the following formula:


$$n\;=\frac{{\left(Z_{1-\alpha -2}+Z_{1-\beta }\right)}^{2}\delta^{2}}{d^{2}}=30$$

### 3.7. Statistical Analysis

The data were analyzed using SPSS for Windows, version 16 (SPSS Inc., Chicago, Ill., USA). Descriptive results are presented as mean ± standard deviation for quantitative variables or as numbers with percentages for qualitative variables. A Friedman test was used to compare changes over time within groups. A Mann–Whitney U test was used to compare outcomes between the two groups at different time intervals. A P-value of < 0.05 was considered statistically significant.

## 4. Results

Thirty patients with a mean age of 61.7 ± 9.2 years were included in the study. The study population comprised 18 males and 12 females. No significant difference was observed between the baseline characteristics of patients in the Triamcinolone group and those in the Magnesium Sulfate group (Table 1). 

**Table 1.** Comparison of the Baseline Characteristics of the Triamcinolone and Magnesium Sulfate Group ^a, b^

Variables	Triamcinolone Group; (n = 15)	Magnesium Sulfate Group; (n = 15)	P-Value
**Age (y)**	62.7 ±10.3	60.73 ± 9.85	0.33
**Gender**			0.48
Male	10 (66.7)	8 (53.3)	
Female	5 (33.3)	7 (46.7)	
**BMI (kg/m** ^**2**^ **)**	28.1 ± 3.9	27. 9 ± (3.7)	0.45
**ASA class**			0.79
I	4 (26.7)	5 (33.3)	
II	11 (73.3)	10 (66.7)	
**Number of involved levels **			0.46
One	9 (60)	10 (66.7)	
Two	6 (40)	5 (33.3)	

Abbreviations: BMI, Body Mass Index; ASA, American Society of Anesthesiologists; VAS, Visual Analogue Scale; ODI, Oswestry Disability Index.

^a^ P < 0.05 is considered statistically significant.

^b^ Values are expressed as mean ± SD or No. (%).

### 4.1. Within-Group Analysis

In the Triamcinolone group, the mean VAS and ODI scores at all time points were significantly lower than the baseline values (P < 0.001). The mean VAS at six months after the intervention was significantly higher than the mean VAS at two weeks and one month after the intervention (P < 0.001). A similar trend was observed in the ODI values, with the mean ODI at three and six months after the intervention being significantly higher than the ODI at one month after the intervention (P < 0.001) (Table 2). 

**Table 2.** Change of Outcome Measures Over Time in the Triamcinolone Group ^a, b^

Measure	Triamcinolone Group; (n=15)	Magnesium Sulfate Group; (n=15)	P-Value
**VAS (baseline)**	7.7 ± 1.1	7.9 ± 0.8	0.63
**VAS (two weeks)**	4.1 ± 1.6	1.1 ± 1	< 0.001
**VAS (one month)**	4.5 ± 1.7	1 ± 0.9	< 0.001
**VAS (three months)**	5.5 ± 1.9	1.2 ± 0.9	< 0.001
**VAS (six months)**	5.9 ± 1.9	2.1 ± 1	< 0.001
**ODI (Baseline)**	49.73 ± 11.3	51.4 ± 9.9	0.39
**ODI (two weeks)**	35.3 ± 15.1	15.3 ± 9.2	< 0.001
**ODI (one month)**	30.7 ± 14.2	13.2 ± 8.4	< 0.001
**ODI (three months)**	35.5 ± 12.7	16.4 ± 7.2	< 0.001
**ODI (six months)**	37.6 ± 11.9	21.6 ± 6.9	< 0.001

Abbreviations: VAS, Visual Analogue Scale; ODI, Oswestry Disability Index.

^a^ P < 0.05 is considered statistically significant.

^b^ Values are expressed as mean ± SD.

A comparable pattern of improvement was observed in the VAS and ODI values of patients in the Magnesium Sulfate group. In this group, the mean VAS and ODI scores at all time points were significantly lower than the baseline values (P < 0.001). The mean VAS at six months after the intervention was significantly higher than the mean VAS at two weeks, one month, and three months after the intervention (P < 0.001). Similarly, the mean ODI at six months after the intervention was significantly higher than the ODI at two weeks, one month, and three months after the intervention (P < 0.001) (Table 2). 

### 4.2. Between-Group Analysis

The mean VAS of the patients before the intervention did not differ significantly between the two study groups. However, after the intervention, the mean VAS at all time points was significantly lower in the Magnesium Sulfate group compared to the Triamcinolone group (P < 0.001) (Table 2). 

**Figure 2.**
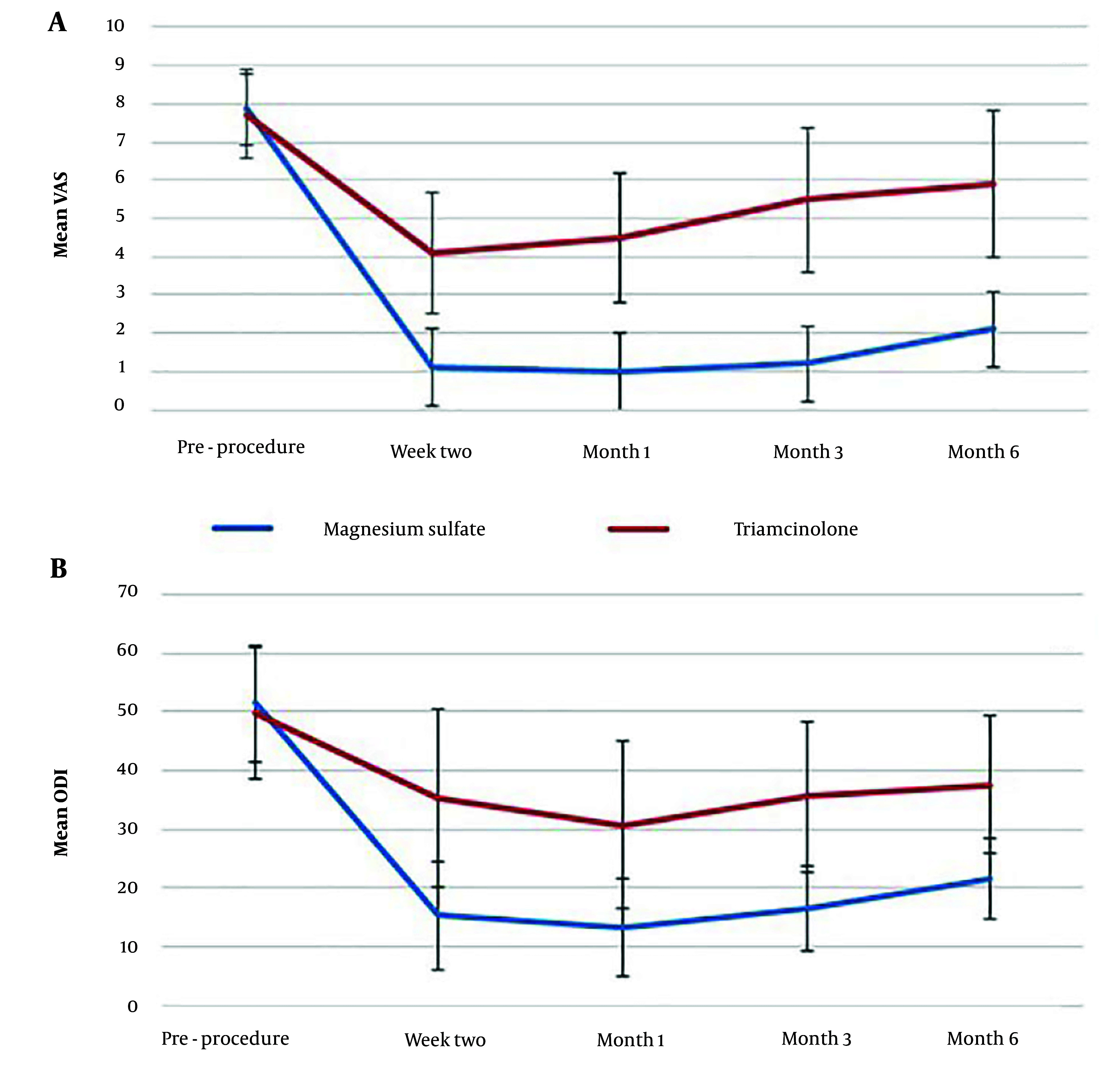
Line charts showing the change of VAS and ODI over time in the two study groups

Similarly, the mean ODI of the patients before the intervention was not significantly different between the two study groups. Following the intervention, the mean ODI at all time points was significantly lower in the Magnesium Sulfate group compared to the Triamcinolone group (P < 0.001) (Table 2). Figure 2 illustrates the changes in VAS and ODI over time for both study groups.

### 4.3. Post-procedure Complications

No serious complications related to the injection were reported by patients in the Triamcinolone group up to the final evaluation. Similarly, no complications were reported by patients in the Magnesium Sulfate group.

## 5. Discussion

In this study, we compared the effects of magnesium sulfate versus triamcinolone epidural injections on pain, function, and adverse effects in the treatment of radicular back pain. Improvement in pain was significantly greater in the magnesium sulfate group. Similarly, functional improvement was significantly better in the magnesium sulfate group. Improvements in pain and function lasted for up to six months in both groups. No significant drug-related adverse effects were reported by the study participants.

Fathy et al., in a randomized controlled trial, compared the effects of transforaminal magnesium sulfate injection and ozone on pain intensity, functional disability, and biomarkers of oxidative stress in 135 patients with symptomatic lumbar disc prolapse. The patients were randomly divided into three groups: Steroids with magnesium sulfate, steroids with ozone, and steroids alone. Pain intensity and functional disability were significantly improved in all groups after two weeks. At one and three months, significant improvements in pain and function were observed only in the magnesium sulfate and ozone groups. At six months, only the magnesium sulfate group continued to show significant improvements in pain and function. Additionally, at two weeks, biomarkers of oxidative stress, including superoxide dismutase (SOD) and glutathione (GSH), significantly increased in both the magnesium sulfate and ozone groups, but not in the steroid-only group. They concluded that transforaminal magnesium sulfate injection provides long-term improvement (up to six months) in pain intensity and functional disability in patients with lumbar disc prolapse (13). Similar results were observed in the present study, where improvement in pain and function was significantly greater in the magnesium sulfate group. However, it should be noted that while we used magnesium sulfate as an alternative to steroids, Fathy et al. used magnesium sulfate in combination with steroids (13).

Awad et al., in a randomized double-blind study, evaluated the efficacy of combining epidural magnesium with steroids (methylprednisolone) for managing lower limb radicular pain (n = 50). Compared to pre-injection measures, pain and function were significantly improved in both groups at all post-injection evaluations (1 day, 1 week, 1 month, and 3 months). Pain and functional scores were significantly better in the combined group compared to the steroid-only group at all post-injection time points (1 day, 1 week, 1 month, and 3 months) (10). They concluded that adding magnesium sulfate to steroids in the transforaminal epidural space could improve pain and function in patients with lower limb radicular pain caused by disc herniation, with this improvement lasting up to 3 months (10). In the present study, magnesium sulfate injection alone, not combined with steroids, resulted in greater improvements in pain and function compared to the steroid-only injection, with benefits lasting up to six months.

Thakur et al., in a "letter to the editor" (14), commented on the study by Awad et al. (10). They pointed out that Awad et al. did not mention the medical management of the patients, which could introduce bias into the interventional study due to heterogeneous medical management. Additionally, Awad et al. did not report side effects associated with transforaminal epidural magnesium sulfate injection. In the present study, we aimed to minimize bias related to medical management by developing a post-injection medication protocol, as detailed in the methods section (10).

The use of magnesium sulfate for other medical conditions, such as cesarean sections, has been reported to be associated with some side effects, including postoperative nausea/vomiting, hypotension, headache, pruritus, shivering, bradycardia, and respiratory issues (15). In the present study, no drug-related side effects were observed in patients from either study group. However, this result might be limited by the small sample size, as evaluating adverse effects, especially rare ones, typically requires a larger sample size (16).

Overall, the results of this study indicate that transforaminal magnesium sulfate injection could be considered an effective and safe alternative to transforaminal steroid injection.

The study had limitations, including the absence of a placebo group, which could introduce bias, and an insufficient sample size, which may limit the generalizability of the findings.

### 5.1. Conclusions

In patients with radicular back pain, transforaminal magnesium sulfate injection provides greater improvement in pain and function compared to transforaminal steroid injection. Therefore, magnesium sulfate injection could be regarded as an effective and safe alternative to transforaminal steroid injection for this patient group.

## Data Availability

The dataset presented in the study is available on request from the corresponding author during submission or after publication.

## Clinical Trial Registration Code:

IRCT201312037984N12.
